# Supplementary material for: Enzymatic kinetic resolution of desmethylphosphinothricin indicates that phosphinic group is a bioisostere of carboxyl group
Source: Commun Chem. 2020 Sep 2;3:121. doi: 10.1038/s42004-020-00368-z (PMC9814759; doi:10.1038/s42004-020-00368-z)
Supplement: Supplementary file 1 — Reporting Summary [file 42004_2020_368_MOESM1_ESM.pdf]

nature research

Corresponding author(s): D. De Biase and A. Khomutov

Last updated by author(s): Aug 2, 2020

# Reporting Summary

Nature Research wishes to improve the reproducibility of the work that we publish. This form provides structure for consistency and transparency in reporting. For further information on Nature Research policies, see our Editorial Policies and the Editorial Policy Checklist.

## Statistics

For all statistical analyses, confirm that the following items are present in the figure legend, table legend, main text, or Methods section.

| n/a | Confirmed | |
|---|---|---|
| ☐ | ☒ | The exact sample size (*n*) for each experimental group/condition, given as a discrete number and unit of measurement |
| ☐ | ☒ | A statement on whether measurements were taken from distinct samples or whether the same sample was measured repeatedly |
| ☒ | ☐ | The statistical test(s) used AND whether they are one- or two-sided<br>*Only common tests should be described solely by name; describe more complex techniques in the Methods section.* |
| ☒ | ☐ | A description of all covariates tested |
| ☒ | ☐ | A description of any assumptions or corrections, such as tests of normality and adjustment for multiple comparisons |
| ☐ | ☒ | A full description of the statistical parameters including central tendency (e.g. means) or other basic estimates (e.g. regression coefficient) AND variation (e.g. standard deviation) or associated estimates of uncertainty (e.g. confidence intervals) |
| ☒ | ☐ | For null hypothesis testing, the test statistic (e.g. *F*, *t*, *r*) with confidence intervals, effect sizes, degrees of freedom and *P* value noted<br>*Give P values as exact values whenever suitable.* |
| ☒ | ☐ | For Bayesian analysis, information on the choice of priors and Markov chain Monte Carlo settings |
| ☒ | ☐ | For hierarchical and complex designs, identification of the appropriate level for tests and full reporting of outcomes |
| ☒ | ☐ | Estimates of effect sizes (e.g. Cohen's *d*, Pearson's *r*), indicating how they were calculated |

*Our web collection on statistics for biologists contains articles on many of the points above.*

## Software and code

Policy information about availability of computer code

| | |
|---|---|
| Data collection | Protein structure was obtained from protein data bank, compounds were drawn by Maestro 2018_2 (Licenced software), Minimizations were run by minimization tool of Maestro GUI; Molecular dynamics were run by Amber 12 (Licenced suite). The molecular dynamics trajectories were visualized by Pymol2.0.<br>For kinetic analysis, the absorbance readings were measured through the UV-Vis ChemStation Software by Agilent Technologies (HP8453).<br>Microplate readings for MIC were collected through either Magellan™ by Tecan or SkanIt™ by ThermoFisher Scientific. |
| Data analysis | The analyses of the MD trajectories were analysed by cpptraj v17.00.<br>NMR spectra were analysed using MestReNova program (https://mestrelab.com) and Bruker Topspin version 3.0.<br>For kinetic analysis, data were analyzed through Microsoft Excel and fitted to the custom Michaelis-Menten equation included in the software Prism - GraphPad version 4.0 and then plotted by using the same software.<br>For MIC analysis, data were analyzed through Microsoft Excel and then plotted by using Prism - GraphPad version 4.0. |

For manuscripts utilizing custom algorithms or software that are central to the research but not yet described in published literature, software must be made available to editors and reviewers. We strongly encourage code deposition in a community repository (e.g. GitHub). See the Nature Research guidelines for submitting code & software for further information.

# Data

Policy information about availability of data

All manuscripts must include a data availability statement. This statement should provide the following information, where applicable:

- Accession codes, unique identifiers, or web links for publicly available datasets
- A list of figures that have associated raw data
- A description of any restrictions on data availability

The data that support the findings of this study are available from the corresponding author upon reasonable request.

# Field-specific reporting

Please select the one below that is the best fit for your research. If you are not sure, read the appropriate sections before making your selection.

[x] Life sciences [ ] Behavioural & social sciences [ ] Ecological, evolutionary & environmental sciences

For a reference copy of the document with all sections, see nature.com/documents/nr-reporting-summary-flat.pdf

# Life sciences study design

All studies must disclose on these points even when the disclosure is negative.

| | |
|---|---|
| Sample size | Sample sizes were chosen based on the standards in the field. |
| Data exclusions | No data were excluded. |
| Replication | Replication was performed based on the standards in the field. |
| Randomization | Randomization was not used in this study. |
| Blinding | Blinding was not used in this study. |

# Reporting for specific materials, systems and methods

We require information from authors about some types of materials, experimental systems and methods used in many studies. Here, indicate whether each material, system or method listed is relevant to your study. If you are not sure if a list item applies to your research, read the appropriate section before selecting a response.

## Materials & experimental systems

| n/a | Involved in the study | |
|---|---|---|
| [x] | [ ] | Antibodies |
| [x] | [ ] | Eukaryotic cell lines |
| [x] | [ ] | Palaeontology and archaeology |
| [x] | [ ] | Animals and other organisms |
| [x] | [ ] | Human research participants |
| [x] | [ ] | Clinical data |
| [x] | [ ] | Dual use research of concern |

## Methods

| n/a | Involved in the study | |
|---|---|---|
| [x] | [ ] | ChIP-seq |
| [x] | [ ] | Flow cytometry |
| [x] | [ ] | MRI-based neuroimaging |
