# Supplementary material for: Enzymatic kinetic resolution of desmethylphosphinothricin indicates that phosphinic group is a bioisostere of carboxyl group
Source: Commun Chem. 2020 Sep 2;3:121. doi: 10.1038/s42004-020-00368-z (PMC9814759; doi:10.1038/s42004-020-00368-z)
Supplement: Supplementary file 2 — Supplementary Information [file 42004_2020_368_MOESM2_ESM.pdf]

Supplementary Information

Enzymatic kinetic resolution of desmethylphosphinothricin indicates that phosphinic group is a bioisostere of carboxyl group

Daniela De Biase[1]*, Francesca Cappadocio[1], Eugenia Pennacchietti[1], Fabio Giovannercole[1], Antonio Coluccia[2], Jouko Vepsalainen[3] and Alex Khomutov[4]*

[1]Department of medico-surgical Sciences and Biotechnologies, Laboratory affiliated to Istituto Pasteur Italia– Fondazione Cenci Bolognetti, Sapienza University of Rome, Corso della Repubblica 79, I-04100 Latina, Italy;

[2]Department of Chemistry and Technology of Drugs, Laboratory affiliated to Istituto Pasteur Italia-Fondazione Cenci Bolognetti, Sapienza University of Rome , Piazzale Aldo Moro 5, I-00185 Roma, Italy;

[3]School of Pharmacy, Biocenter Kuopio, University of Eastern Finland, Kuopio Campus, P.O. Box 1627 Kuopio, FI-70211 Finland;

[4]Engelhardt Institute of Molecular Biology, Russian Academy of Sciences, Vavilov St., 32, Moscow, 119991, Russia.

* Corresponding authors:

Daniela De Biase, Department of Medico-Surgical Sciences and Biotechnologies, Sapienza University of Rome, Corso della Repubblica 79, 04100 Latina, ITALY, Tel.: +39-0773-1757212; E-mail: daniela.debiase@uniroma1.it;

Alex Khomutov, Engelhardt Institute of Molecular Biology, Russian Academy of Sciences, Vavilov St., 32, Moscow, 119991, RUSSIA, Tel.: +7-499-1356065; E-mail: alexkhom@list.ru.

Contents

Supplementary Fig. 1. Phosphinic, but not phosphonic group, as bioisoster of carboxyl group 2
Supplementary Fig. 2. TLC analysis of *Ec*GadB reaction mixture with *D,L*-Glu-γ-P_H and *D,L*-Glu-γ-P as substrates 3
Supplementary Fig. 3. ^{1}H-NMR monitoring of the decarboxylation of *D,L*-Glu-γ-P_H by *Ec*GadB 4
Supplementary Fig. 4. Time course of GABA-P_H formation (as assessed by Gabase) following the decarboxylation of *D,L*-Glu-γ-P_H by *Ec*GadB 5
Supplementary Fig. 5. ^{1}H, ^{13}C, and ^{31}P NMR spectra of GABA-P_H 6
Supplementary Fig. 6. Reaction of PLP with aminooxyethyl putrescine (AOEPUT) 7
Supplementary Fig. 7. ^{1}H, ^{13}C, and ^{31}P NMR spectra of *D*-Glu-γ-P_H 8
Supplementary Methods 9
Supplementary References 13

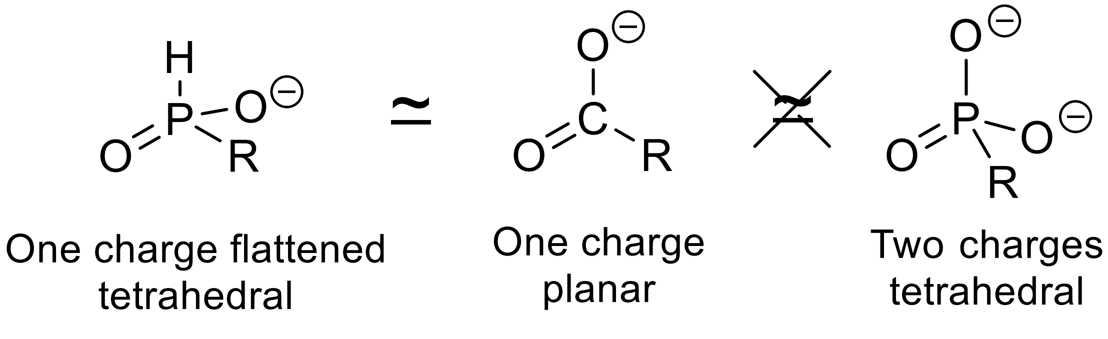

Supplementary Figure 1. Phosphinic (on the left), but not phosphonic (on the right) group, as bioisoster of carboxyl group (in the middle) as suggested by crystallographic evidence on the different bond lengths in the β-phosphinic analogue of aspartate. [1]

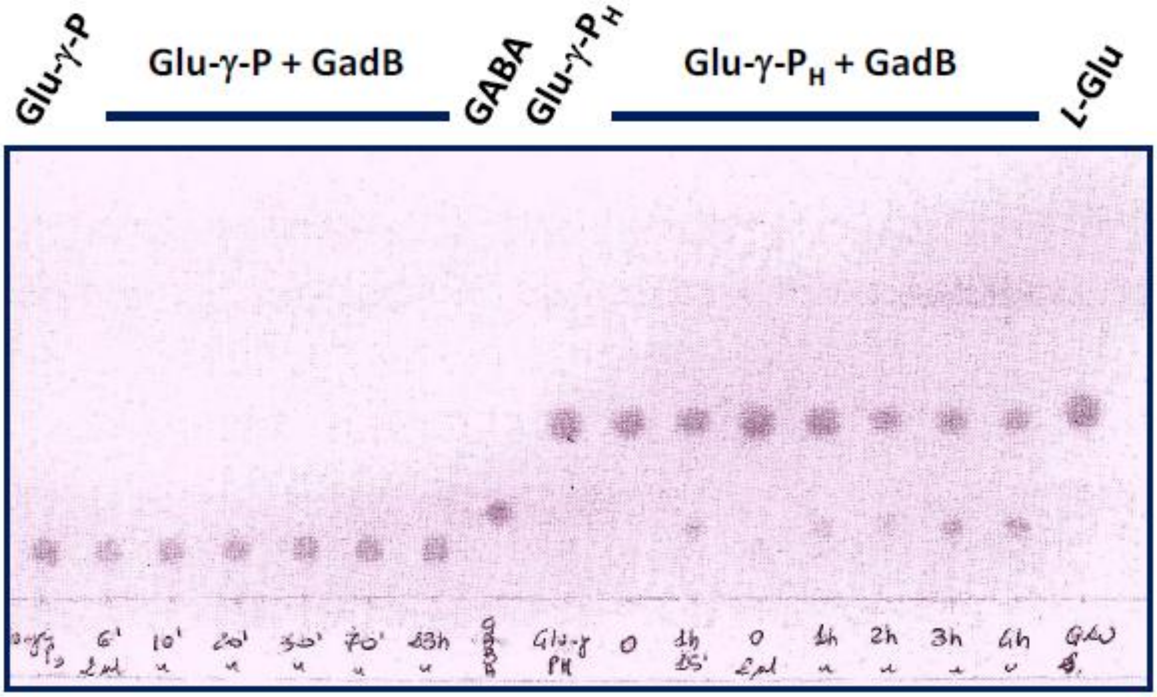

Supplementary Figure 2. TLC analysis of *Ec*GadB reaction with Glu-γ-P_H (right) and Glu-γ-P (left; the γ-phosphonic counterpart of Glu-γ-P_H) as substrates. The reactions were carried out in a final volume of 220 µL using 18 mM of each substrate. The buffer used was 10 mM sodium acetate buffer, pH 4.6. *Ec*GadB was 160 µg. Standards (20 nmoles): Glu-γ-P, Glu-γ-P_H, *L*-Glu and GABA. Staining was with ninhydrin. TLC was carried out on silica plates (Merck, Germany) in *i*-PrOH:25%NH_4OH:H_2O, 7:1:2 v/v.

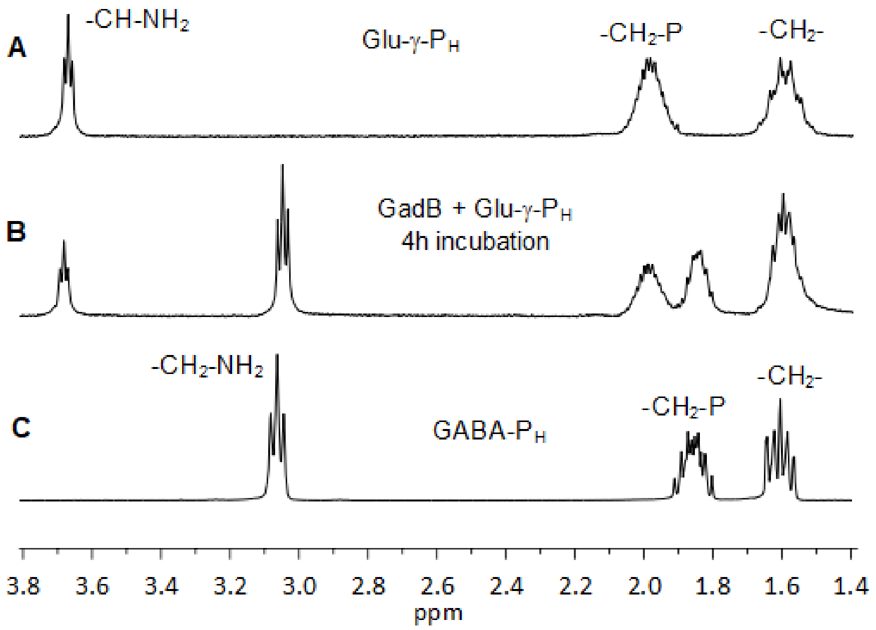

Supplementary Figure 3. ^{1}H-NMR monitoring of the enzymatic decarboxylation of *D,L*-Glu-γ-P_H by GadB. (**A**) fragment of ^{1}H-NMR spectrum of GadB substrate mixture (zero time); (**B**) fragment of ^{1}H-NMR spectrum of the substrate mixture (4 h); (**C**) fragment of ^{1}H-NMR spectrum of GABA-P_H. The *Ec*GadB decarboxylation reaction of Glu-γ- P_H (18 mM) was carried out at 37°C in 0.1 M Pyridine/HCl buffer, pH 4.6, and the pH adjusted with small additions of diluted HCl. The reaction was halted after 4 hours by removing *Ec*GadB by ultrafiltration. *Ec*GadB was 0.09 mg mL^{-1} and the reaction volume 1 ml.

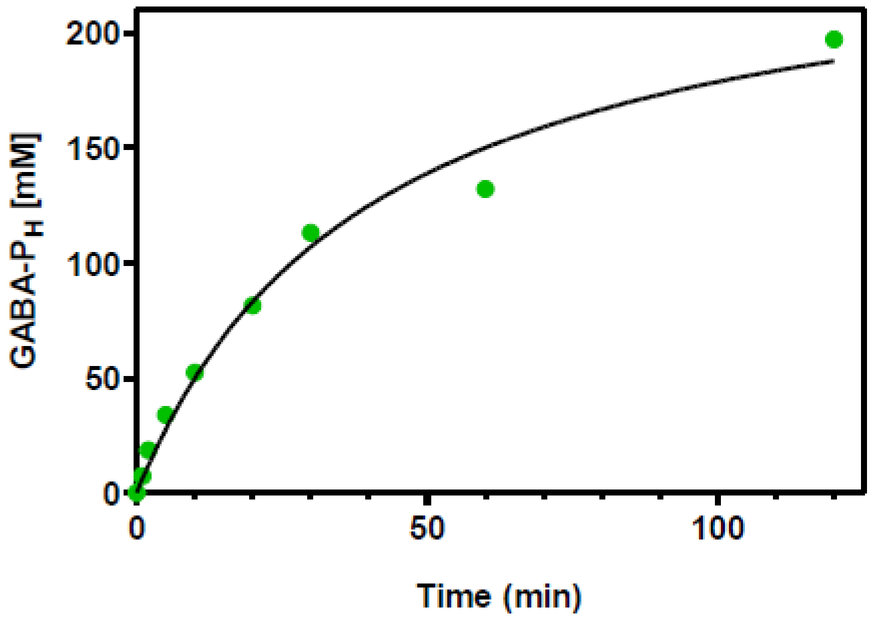

Supplementary Figure 4. Time course of GABA-P_H formation (as assessed by GABase) following the decarboxylation of *D,L*-Glu-γ-P_H by *Ec*GadB. The reaction (100 µL) was performed at 37°C in 200 mM Pyridine/HCl buffer, pH 4.6, containing 1 mM PLP, 0.1 mM DTT, 0.4 M of *D,L*-Glu-γ-P_H to which *Ec*GadB was added to a final concentration of 1 µg μL^{-1}. At time intervals (0, 2, 5, 10, 20, 30, 60, 120 minutes), aliquots (5 µL) were transferred in 50 µL of 10 mM NaOH and then 2 µL analyzed for GABA-P_H content with the GABase assay as described in Experimental section During the reaction, the pH was periodically adjusted to the value 4.6-5.0 by stepwise addition of 1 N HCl (total 10 µL). A representative experiment is shown.

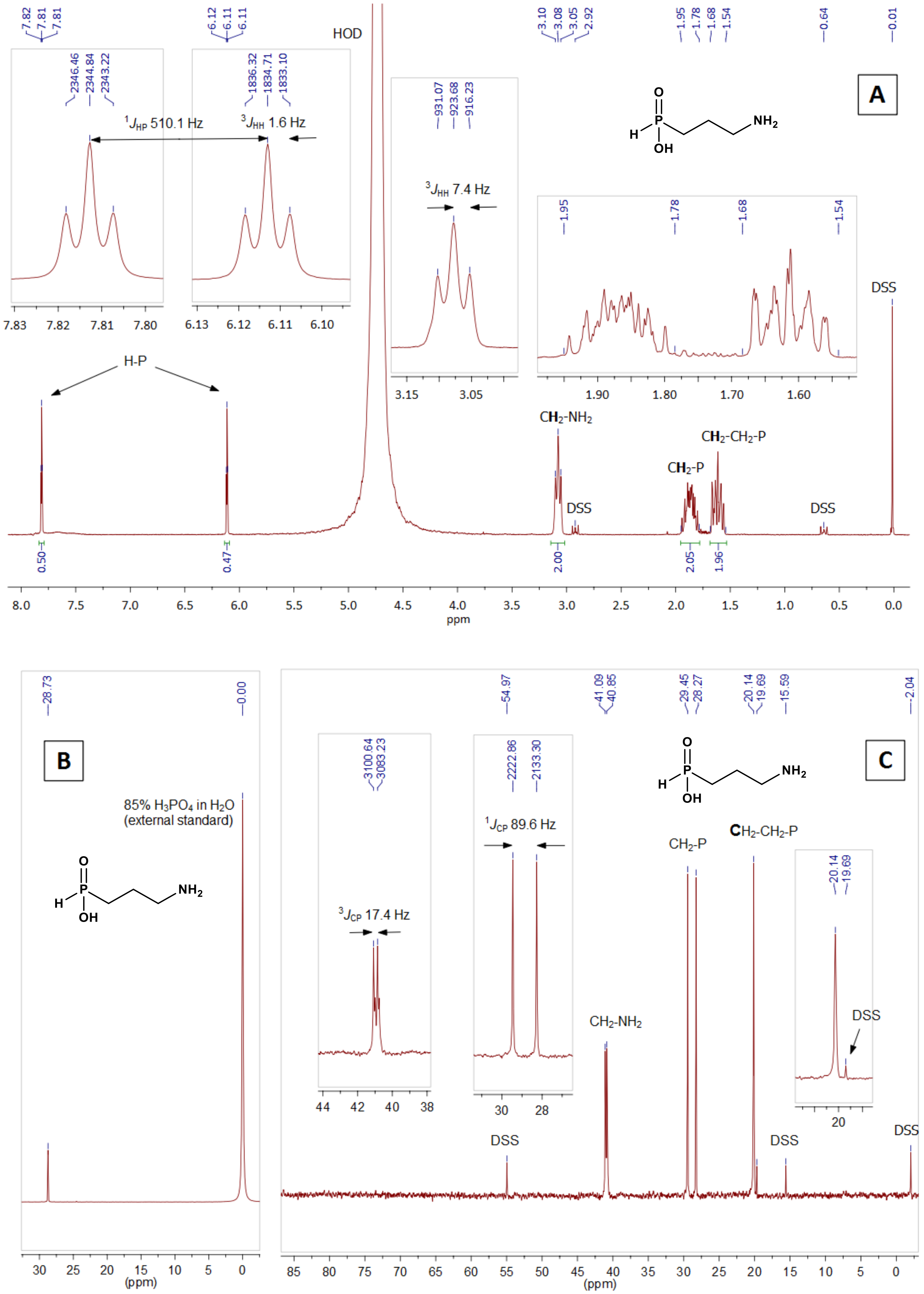

Supplementary Figure 5. NMR spectra of GABA-P$_H$. **A:** ^{1}H NMR spectrum in D_2O/H_2O = 1/1; **B:** ^{31}P-decoupled NMR spectrum in D_2O/H_2O = 1/1; **C:** ^{13}C NMR spectrum in D_2O/H_2O = 1/1.

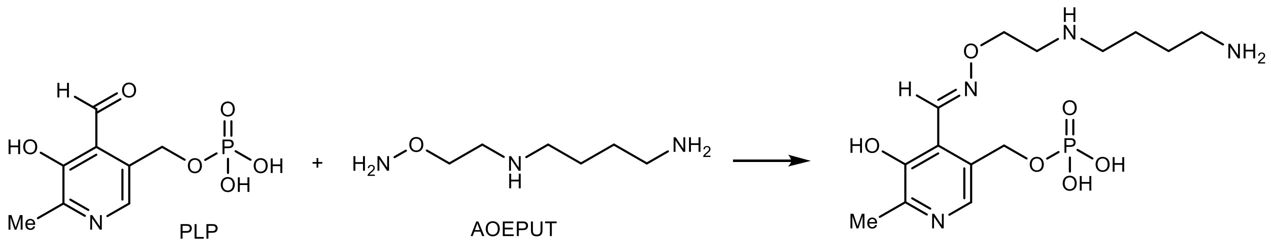

Supplementary Figure 6. Reaction of PLP with aminooxyethyl putrescine (AOEPUT)

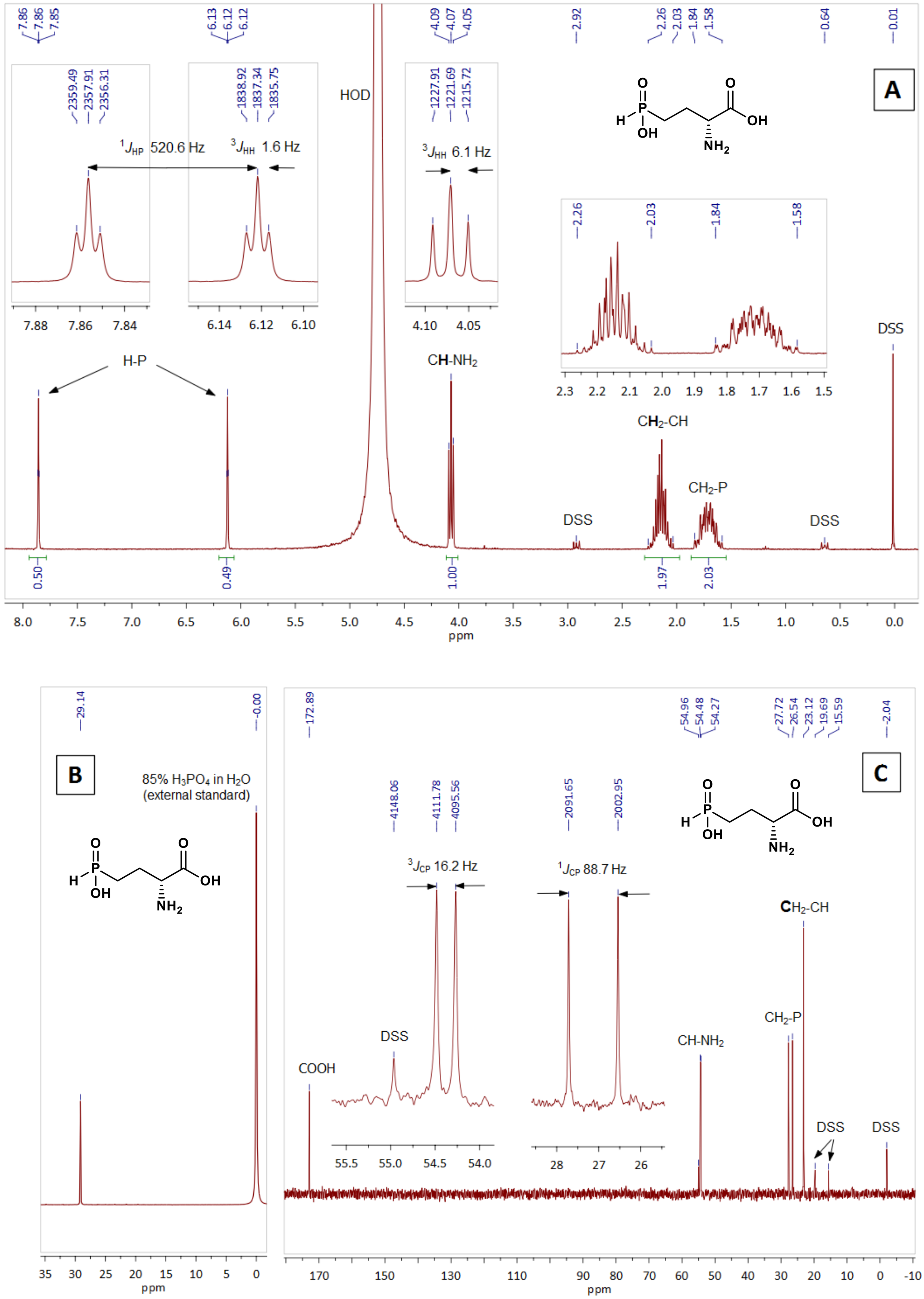

Supplementary Figure 7. NMR spectra of *D*-Glu-γ-P_H. **A:** ^1H NMR spectrum in D_2O/H_2O = 9/1; **B:** ^{31}P-decoupled NMR spectrum in D_2O/H_2O = 1/1; **C:** ^{13}C NMR spectrum in D_2O/H_2O = 1/1.

Supplementary Methods

Expression and Purification of *E. coli* GadB (*Ec*GadB).

Overexpression and purification of recombinant wild type *Ec*GadB were carried out essentially as previously described [2], except that the column chromatography was on a pre-packed HiPrep DEAE FF 16/10 (20 mL) as reported elsewhere. [3] Protein purity was assessed by 12% SDS-PAGE and by measuring the PLP content which was determined by treating an aliquot of *Ec*GadB with 0.1 N NaOH and then measuring the absorbance of the cofactor free in solution at 388 nm (applying the molar absorption coefficient (ε) PLP_{388}= 6550 $M^{-1}cm^{-1}$). [4] Enzyme concentration and activity were measured as previously described.[2,5] *Ec*GadB UV-visible spectra were recorded at controlled temperature on a Hewlett–Packard Agilent model 8453 diode array spectrophotometer.

Synthesis of *L*-2-Amino-4-(hydroxyphosphinyl)butyric Acid (*L*-DMPT, *L*-Glu-γ-P_H).

L-2-(Benzyloxycarbonyl)amino-3-butenoic acid methyl ester (methyl *N*-Cbz-*L*-vinylglycine) was from Ark Pharm Inc. (IL, USA) and purified from contaminating *L*-2-(bezyloxycarbonyl)amino-2-butenoic acid methyl ester by column chromatography on Kieselgel 60 (40 – 63 μm, Merck, Germany), using for elution hexane:EtOAc, 4:1 v/v. All other reagents and solvents were highest purity and used as supplied by Aldrich and Acros.

TLC was carried out on Kieselgel 60 F_{254}, or Cellulose F_{254} plates (Merck, Germany) in the indicated solvent systems.. Aminophosphinates were detected on TLC plates following staining with ninhydrin (0.4% in acetone) and with ammonium molibdate reagent, while Cbz-derivatives were detected by UV absorbance and ammonium molibdate reagent.

Ion-exchange chromatography was carried out on Dowex 50Wx8, H^+-form, 100-200 mesh (BioRad, USA) using water for elution.

NMR spectra were recorded on a Bruker AM-300 instrument in D_2O, or in D_2O/H_2O mixtures with sodium 3-trimethyl-1-propanesulfonate as internal, or 85% H_3PO_4 as external standards. Chemical shifts are given in ppm.

High resolution mass spectra (HR MS) were measured on a Bruker micrOTOF II instrument using electrospray ionization (ESI). [6] The measurements were done in a negative ion mode (interface capillary voltage – 2000 V); mass range from *m/z* 50 to *m/z* 1050; internal calibration was done with ESI Tuning Mix, Agilent. A syringe injection was used for *L*- and *D*-Glu-γ-P_H in $CH_3CN:H_2O$, 50:50 v/v solutions (flow rate 5 $\mu L \cdot min^{-1}$). Nitrogen was applied as a dry gas and the interface temperature was set at 180°C.

Optical rotations were recorded on a 341 Polarimeter (Perkin-Elmer), solvents and concentrations are indicated in the text.

Melting points were determined in open capillary tubes on Electrothermals Mel-Temp 1202D instrument and are uncorrected.

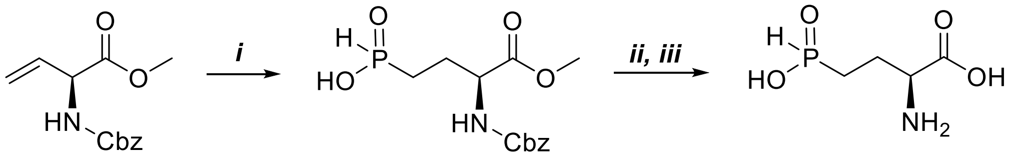

i- H_3PO_2/AIBN/MeOH/Δ; ***ii***- HCl/H_2O/Δ; ***iii***- Dowex 50x8 (H^+)

A mixture of hypophosphorous acid (H_3PO_2, 2.85 g, 21.5 mmol, 50% aqueous), methyl *N*-Cbz-*L*-vinylglycine (595 mg, 2.4 mmol), and α,α'-azoisobutyronitrile (AIBN, 36 mg, 0.22 mmol) in MeOH (10 mL) was refluxed in Ar atmosphere for 5 h. Then water (10 mL) was added, MeOH was evaporated *in vacuo,* and the residue was extracted with EtOAc (3 x 5 mL). The EtOAc solution was concentrated *in vacuo*, the residue dissolved in 5 mL of solvent system A and methyl *L*-2-(*N*-benzyloxycarbonyl)amino-4-(hydroxyphosphinyl)butanoate was purified by column chromatography on Kieselgel (65 g, 3.5 x 11 cm) eluting with solvent system A. Fractions

containing target phosphinate (from 225 ml to 305 mL) were combined, evaporated to dryness *in vacuo* and the residue was dried *in vacuo* over P_2O_5 to give pure methyl *L*-2-(*N*-benzyloxycarbonyl)amino-4-(hydroxyphosphinyl)butanoate. TLC (Kieselgel 60 F_{254}, dioxane:25%NH_4OH:H_2O, 85:15:5 v/v); R_f = 0.29.

The resulting methyl *L*-2-(*N*-benzyloxycarbonyl)amino-4-(hydroxyphosphinyl)butanoate was refluxed in 20% aq. HCl (25 mL) in Ar atmosphere for 5 h and extracted with Et_2O (3 x 8 mL). Water phase was concentrated *in vacuo*, co-evaporated *in vacuo* with water (3 x 10 mL) and the residue was purified using Dowex 50WX8 column (100-200 mesh, H^+-form, V = 35 mL). Fractions containing *L*-Glu-γ-P_H (from 160 mL to 400 mL) were combined, evaporated to dryness *in vacuo*, the residue was recrystallized from water/EtOH and dried *in vacuo* over P_2O_5 to give pure *L*-Glu-γ-P_H (166 mg, 42%, as calculated for methyl *N*-Cbz-*L*-vinylglycine). mp 220-221°C, dec. (lit.[7] 221-222°C, dec.); TLC (Cellulose F_{254}, *i*-PrOH:25%NH_4OH:H_2O, 7:1:2 v/v); R_f = 0.24; $[\alpha]_D^{20}$ = +20.9 (c=1 in H_2O) [lit.[7] $[\alpha]_D^{20}$ +7.9 (c=0.4 in H_2O); lit.[8] $[\alpha]_D^{20}$ = +28.9 (c=1 in 1 N HCl)]; ^1H NMR (300 MHz, D_2O) δ: 6.98 (dt, $^1J_{HP}$ = 520.6 Hz, $^3J_{HH}$ = 1.6 Hz, 1H, P-H), 4.07 (t, $^3J_{HH}$ = 6.1 Hz, 1H,CH), 2.26-2.03 (m, 2H, C$\underline{H}_2$CH), 1.84-1.58 (m, 2H, C$\underline{H}_2$P). ^{13}C NMR (75.5 MHz, D_2O) δ: 174.9 s, 54.4 d ($^3J_{CP}$ = 16.2 Hz), 27.1 d ($^1J_{CP}$ = 88.9 Hz), 23.1 s. ^{31}P NMR (121.5 MHz, D_2O) δ: 29.14 [lit.[7] ^{31}P NMR (D_2O) δ: 39.91]; HRESIMS (m/z): $[M–H]^-$ calcd. for $C_4H_9NO_4P^-$, 166.0275; found, 166.0275.

Isolation of *D*-Glu-γ-P_H and GABA-P_H by ion-exchange chromatography.

After allowing the *Ec*GadB reaction to proceeded to ≥ 98% completion (as assessed by NMR), the enzyme was separated with ultrafiltration (Vivaspin devices) and to the filtrate (2.2-2.3 mL) MilliQ water was added (7.5 mL). This solution was applied on a Dowex 50WX8 column (V= 20 mL) and eluted with 400 mL of H_2O. Fractions containing *D*-Glu-γ-P_H (from 76 mL to 235 mL) were combined and evaporated to dryness *in vacuo*. This resulted in solid *D*-Glu-γ-P_H, which was slightly yellow due to the contaminations of PLP originally present in the *Ec*GadB reaction mixture.

The elution was continued with 0.5 N HCl (300 mL) and GABA-P_H containing fractions (from 40 mL to 130 mL) were combined, evaporated to dryness *in vacuo*, co-evaporated *in vacuo* with H_2O (4 x 5 mL). The residue was dissolved in a minimal volume of EtOH and propylene oxide was added dropwise until precipitation started. To the obtained suspension an equal volume of *i*-PrOH was added and the mixture was left at +4°C until complete precipitation. The solid material was filtered off, recrystallized from H_2O/EtOH and dried *in vacuo* over P_2O_5 to give GABA-P_H (0.53 g, 60 % based on *L*-isomer content in *D,L*-Glu-γ-P_H): mp 208-211°C (lit.[9] 209-213°C); TLC (Cellulose F_{254}, *i*-PrOH:25%NH_4OH:H_2O, 7:1:2, v/v); R_f = 0.42; ^{1}H-NMR (300 MHz, D_2O) δ: 6.96 (dt, $^1J_{HP}$ = 510.1 Hz, $^3J_{HH}$ = 1.6 Hz, 1H, P-H), 3.08 (t, $^3J_{HH}$ = 7.4 Hz, 1H, $C\underline{H}_2NH_2$), 1.95-1.78 (m, 2H, CH_2P), 1.68-1.54 (m, 2H, $C\underline{H}_2CH_2NH_2$). ^{13}C-NMR (75.5 MHz, D_2O) δ: 40.97 d ($^3J_{CP}$ = 17.4 Hz), 28.9 d ($^1J_{CP}$ = 89.8 Hz), 20.14 s. ^{31}P-NMR (121.5 MHz, D_2O) δ: 28.73. The ^{1}H, ^{13}C and ^{31}P NMR spectra of GABA-P_H are shown in Supplementary Figure 5.

Slightly yellow *D*-Glu-γ-P_H, contaminated with PLP, was allowed to react with AOEPUT trihydrochloride (2.6 mg, 0.01 mmol) in H_2O (5 mL) as depicted at Supplementary Figure 6. The pH of the reaction mixture was adjusted to pH ~ 4.5-5.0 using diluted aq. NH_4OH, the reaction mixture was incubated for 2 h at 20°C, applied on Dowex 50WX8 column (V = 8 mL) and eluted with 150 mLH_2O. Fractions containing *D*-Glu-γ-P_H (from 31 mL to 105 mL) were combined and evaporated to dryness *in vacuo*. The residue was crystallized from H_2O/EtOH and after drying *in vacuo* over P_2O_5 it yielded *D*-Glu-γ-P_H (61 mg, 73 % based on *D*-isomer content in *D,L*-Glu-γ-P_H): $[\alpha]_D^{20}$ -20.4° (c=1, H_2O); mp 222-223°C, dec. [lit. [8]: 221-222°C, dec. (*L*-isomer)]; TLC (Cellulose F_{254}, *i*-PrOH:25%NH_4OH:H_2O, 7:1:2, v/v): R_f = 0.24;. $[\alpha]_D^{20}$ -20.4° (c=1 in H_2O); ^{1}H-NMR (300 MHz D_2O) δ: 6.99 (dt, $^1J_{HP}$ = 517.7 Hz, $^3J_{HH}$ = 1.6, 1H, P-H), 4.08 (t, $^3J_{HH}$ = 6.0 Hz, 1H, CH), 2.26-2.03 (m, 2H, $C\underline{H}_2CH$), 1.84-1.59 (m, 2H, $C\underline{H}_2P$). ^{13}C-NMR (75.5 MHz, D_2O) δ: 172.9 s, 54.4 d ($^3J_{CP}$ = 16.2 Hz), 27.1 d ($^1J_{CP}$ = 88.7 Hz), 23.1 s. ^{31}P-NMR (121.5 MHz, D_2O) δ: 29.14; HRESIMS

(m/z): $[M–H]^-$ calcd. for $C_4H_9NO_4P^-$, 166.0275; found: 166.0273. The ^{1}H, ^{13}C and ^{31}P NMR spectra of *D*-Glu-γ-P_H are presented in Supplementary Figure 7.

Supplementary References

1 Schwalbe, C. H. W., Freeman, S. & DasGupta, M. 2-Amino-2-carboxyethylphosphinic acid monohydrate. *Acta Crystallographica Section C* **49**, 1826-1828, doi:10.1107/s0108270193003683 (1993).

2 De Biase, D., Tramonti, A., John, R. A. & Bossa, F. Isolation, overexpression, and biochemical characterization of the two isoforms of glutamic acid decarboxylase from Escherichia coli. *Protein expression and purification* **8**, 430-438 (1996).

3 Giovannercole, F. *et al.* On the effect of alkaline pH and cofactor availability in the conformational and oligomeric state of Escherichia coli glutamate decarboxylase. *Protein engineering, design & selection : PEDS* **30**, 235-244, doi:10.1093/protein/gzw076 (2017).

4 Peterson, E. A. & Sober, H. A. Preparation of crystaline phosphorilated derivatives of vitamin B6. *Journal of American Chemical Society* **76**, 169-175 (1954).

5 Grassini, G., Pennacchietti, E., Cappadocio, F., Occhialini, A. & De Biase, D. Biochemical and spectroscopic properties of Brucella microti glutamate decarboxylase, a key component of the glutamate-dependent acid resistance system. *FEBS open bio* **5**, 209-218, doi:10.1016/j.fob.2015.03.006 (2015).

6 Tsedilin, A. M. *et al*. How sensitive and accurate are routine NMR and MS measurements? *Mend. Commun*. **25**, 454–456, doi:10.1016/j.mencom.2015.11.019 (2015).

7 Selvam, C., Goudet, C., Oueslati, N., Pin, J. P. & Acher, F. C. L-(+)-2-Amino-4-thiophosphonobutyric acid (L-thioAP4), a new potent agonist of group III metabotropic glutamate receptors: increased distal acidity affords enhanced potency. *Journal of medicinal chemistry* **50**, 4656-4664, doi:10.1021/jm070400y (2007).

8 Seto, H. *et al.* Studies on the biosynthesis of bialaphos (SF-1293). 2. Isolation of the first natural products with a C-P-H bond and their involvement in the C-P-C bond formation. *The Journal of antibiotics* **36**, 96-98 (1983).

9 Dingwall, J. G., Ehrenfreund, J. & Hall, R. G. Diethoxymethylphosphonites and phosphinates. Intermediates for the synthesis of α,β- and X aminoalkylphosphonous acids. *Tetrahedron* **45**, 3787-3808, doi:https://doi.org/10.1016/S0040-4020(01)89240-1 (1989).
